# Supplementary material for: Splice-Junction-Based Mapping of Alternative Isoforms in the Human Proteome
Source: Cell Rep. Author manuscript; Available in PMC 2020 Jan 15. (PMC6961840; doi:10.1016/j.celrep.2019.11.026)
Supplement: 2 [file NIHMS1546469-supplement-2.zip › DF1/PXD010154/Prostate/Prostate_14_MINDY3_LYSLNNCILLK.pdf]

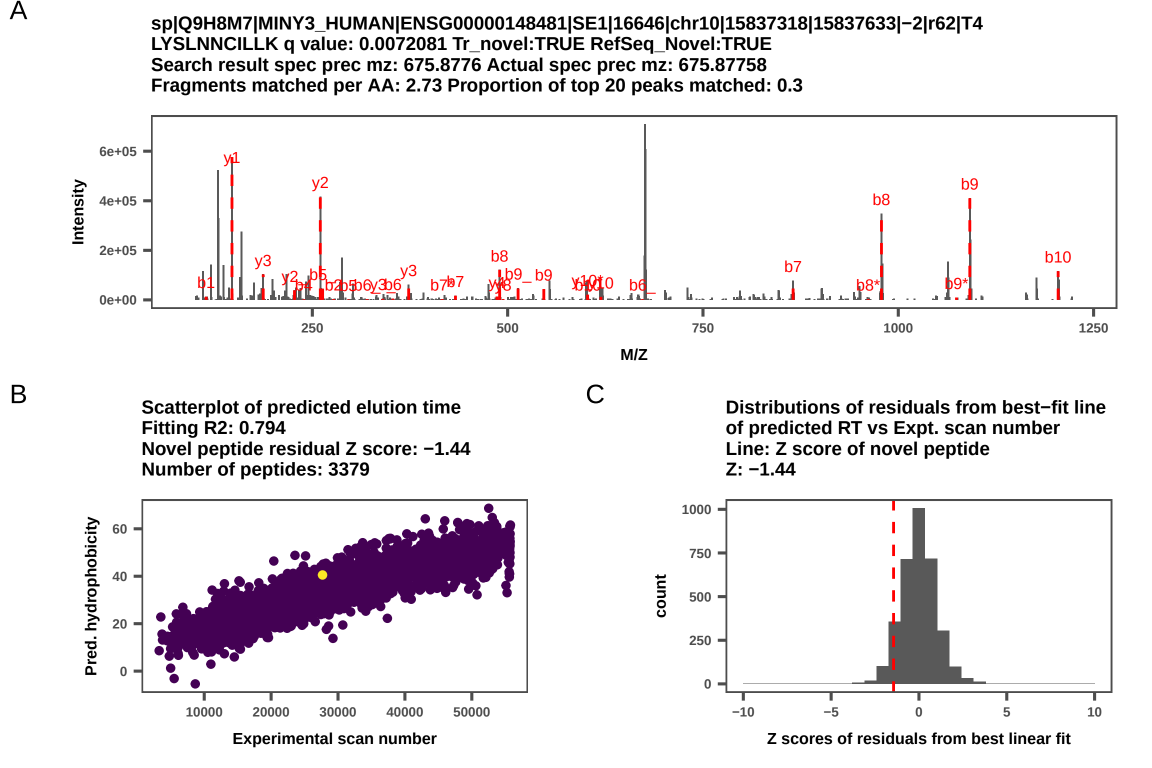
A
sp|Q9H8M7|MINY3_HUMAN|ENSG00000148481|SE1|16646|chr10|15837318|15837633|−2|r62|T4
LYSLNNCILLK q value: 0.0072081 Tr_novel:TRUE RefSeq_Novel:TRUE
Search result spec prec mz: 675.8776 Actual spec prec mz: 675.87758
Fragments matched per AA: 2.73 Proportion of top 20 peaks matched: 0.3
Intensity
6e+05
4e+05
2e+05
0e+00
M/Z
250
500
750
1000
1250
y1
y2
y3
b1
b8
b9
b7
b10
b8*
b9*
B
Scatterplot of predicted elution time
Fitting R2: 0.794
Novel peptide residual Z score: −1.44
Number of peptides: 3379
Pred. hydrophobicity
60
40
20
0
10000
20000
30000
40000
50000
Experimental scan number
C
Distributions of residuals from best−fit line
of predicted RT vs Expt. scan number
Line: Z score of novel peptide
Z: −1.44
count
1000
750
500
250
0
−10
−5
0
5
10
Z scores of residuals from best linear fit
